# Implantable venous access port placement in the upper arm of breast cancer patients with persistent left superior vena cava: a case series and literature review

**DOI:** 10.3389/fcvm.2026.1792914

**Published:** 2026-04-10

**Authors:** Wei Gao, Xiaochen Zhang, Songying Zhu, Huayu Bai, Ran Tan, Ning Zhang

**Affiliations:** Department of Breast Surgery, Qilu Hospital of Shandong University, Jinan, Shandong, China

**Keywords:** breast cancer, implantable venous access port, intracavitary electrocardiogram, P wave, persistent left superior vena cava

## Abstract

Persistent left superior vena cava (PLSVC) is a rare congenital venous anomaly. Although implantable venous access port placement in PLSVC has been reported, the optimal technique, catheter positioning, and safety considerations remain uncertain. This study describes our experience in managing three breast cancer patients with PLSVC and proposes a safe and efficient approach for port placement. All three cases of PLSVC were identified among breast cancer patients undergoing implantable venous access port placement guided by intracavitary electrocardiogram (IC-ECG). A negative P wave appeared on IC-ECG, and persisted as it advanced toward the predicted insertion length. These findings were reproducible on repeated catheterizations. To avoid excessive tip depth, the catheter was withdrawn by approximately 3 cm from the predetermined length. Postoperative X-ray and CT confirmed the final tip position at the T6 level. No functional impairment or catheter-related complications occurred during the entire course of treatment. Therefore, when placing a port from the left side, the presence of a negative P wave upon reaching the predetermined length may indicate PLSVC. Persistence of the negative P wave during further advancement, or the emergence of bidirectional P waves, should heighten suspicion for this anomaly. Postoperative X-ray and CT can confirm both the presence of PLSVC and the final tip position. Based on observed left-right predetermined length discrepancies in the normal population, withdrawing the catheter by approximately 3 cm from the predicted insertion length provides a useful reference. However, final confirmation of tip location should always rely on imaging.

## 1 Introduction

Persistent left superior vena cava (PLSVC) is a rare congenital vascular anomaly, with an estimated prevalence of 0.2-3.0% in the general population, rising to 1.3-11.0% among individuals with congenital heart disease (1). Except in cases involving a right-to-left shunt, PLSVC is typically asymptomatic and is most often detected incidentally during procedures such as implantation of cardiac devices (2, 3), hemodialysis catheters (4, 5), or central venous catheters (6, 7). While this anatomical variant usually does not affect quality of life, it can complicate certain surgical and interventional procedures.

An implantable venous access port (IVAP) is essential for the safe and effective delivery of systemic anticancer therapy, particularly in breast cancer patients requiring long-term chemotherapy (8). To minimize the risk of upper limb lymphedema after axillary lymph node dissection, ports are usually placed contralateral to the surgical side. For patients with right breast cancer, this often leaves the left upper limb as the only feasible access site. Because catheterization via the left upper arm increases the likelihood of encountering PLSVC, its presence becomes a critical procedural consideration in this population, as access into the anomalous vessel is inevitable.

Intracavitary electrocardiography (IC-ECG) is widely used for guiding central venous catheter tip positioning, relying on P wave changes. However, literature describing IC-ECG characteristics in PLSVC is scarce, and practical guidance for safe management in such cases is limited. Here, we report three cases with right breast cancer and PLSVC who underwent left upper limb venous access port implantation under IC-ECG guidance. We detail the intraoperative IC-ECG findings, surgical technique, and postoperative imaging, and review the literature to provide practical recommendations for managing catheterization in PLSVC.

## 2 Case series

This report describes three female patients who underwent chemotherapy following right breast cancer surgery. The study period was October 2022 to March 2024. PLSVC was identified intraoperatively via IC-ECG during venous access port insertion and confirmed postoperatively by X-ray and CT imaging. None of the patients had significant comorbidities or prior surgical history, and all preoperative assessments were unremarkable. The catheters remained in place for the entire chemotherapy course (mean retention 189 days; range 175–209 days) without functional impairment or catheter-related complications. Patient demographics and follow-up data are summarized in Table 1.

**Table 1:** The general data of patients with PLSVC and their ports published in the last ten years.

Source	Year	Age	Gender	Primary disease	Usage	Identification	Superior vena cava	Type	Retention time (day)	Catheter depth (cm)	Catheter diameter (Fr)	Puncture vein	Tip position	CS diameter (mm)	Complication in use
Our unit	2022	44	Female	Breast cancer	Ch	IC-ECG	Double SVC	I	209	37	5	BV	T6	11	None
	2023	54	Female	Breast cancer	Ch	IC-ECG	Double SVC	I	183	35	5	BV	T6	21	None
	2023	54	Female	Breast cancer	Ch	IC-ECG	Double SVC	I	175	34	5	AV	T6		None
Z. W. et al. (26)	2024	51	Female	Breast cancer	Ch	X-ray	Double SVC	I		23	6.6	IJV	T7	20	None
X. W. et al. (10)	2023	48	Female	Breast cancer	Ch	IC-ECG	RSVC atresia		537	16	5	IJV			None
		71	Female	Rectal cancer	Ch	IC-ECG	RSVC atresia		156	17	5	IJV (right)			None
S. M. et al. (28)	2023	85	Female	cerebral infarction	parenteral nutrition	X-ray	Double SVC		0		7.5	SV			
R. Z. et al. (35)	2022	40	Female	Breast cancer	Ch	X-ray	Double SVC	I			6	IJV	T7		None
		45	Female	Breast cancer	Ch	X-ray	Double SVC	I			6	IJV	T7		None
Y. J. et al. (21)	2021	46	Female	Breast cancer	Ch	IC-ECG			136	25	6.6				None
		41	Female	Breast cancer	Ch	IC-ECG			96	21	6.6				None
		33	Female	Breast cancer	Ch	IC-ECG			756	24	6.5				None
		31	Female	Breast cancer	Ch	IC-ECG			465	20	6.6				None
		59	Male	NSCLC	Ch	IC-ECG			558	21	6.6				None
		57	Female	Breast cancer	Ch	IC-ECG			168	25	6.6				None
		41	Male	Breast cancer	Ch	IC-ECG			240	21	7				None
		51	Female	Breast cancer	Ch	IC-ECG			167	19	7				None
J. V. W. et al. (9)	2018	74	Male	NSCLC	Ch	venography	Double SVC	I				SV			
E. A. K. et al. (36)	2016	63	Male	colon cancer		Contrast enhanced CT	Double SVC		0	45		BV			

NSCLC, nonsmall cell lung cancer; BV, basilic vein; Ch, Chemotherapy; SV: subclavian vein; IJV: internal jugular vein; AV: axillary vein.

## 3 Procedures

All patients had right breast cancer and underwent left upper arm venous access port implantation. Patients were positioned supine with the left arm abducted at 90°. The circumference of the left upper limb was measured. The basilic or axillary vein was selected under ultrasound guidance, and the puncture site was marked. Catheter length was pre-determined by summing the body surface distances from the puncture site to the acromion, the acromion to the sternoclavicular joint, and from the sternoclavicular joint to the third intercostal space along the sternal border.

After sterile preparation and local anesthesia with 2% lidocaine, ultrasound-guided venous puncture was performed. Upon confirming dark venous blood return, the puncture needle was exchanged for a guidewire. A tearable sheath was advanced over the guidewire, which was then removed. The catheter with a supporting guidewire was introduced to the predetermined length. Connecting the guidewire to the IC-ECG electrode generate P waves tracings, which were recorded as the catheter was advanced cautiously. When PLSVC was suspected, the catheter was retracted approximately 3 cm according to the predetermined length to avoid overly deep tip placement. The tearable sheath was withdrawn; a skin incision was made 2–3 cm below the puncture site, and subcutaneous tissue bluntly dissected to create a pouch for the port body. The subcutaneous tunnel was established, catheter length confirmed, and the port connected. Blood aspiration and pulse flushing verified catheter patency. The incision was closed in layers, dressed sterilely, and pressure applied.

## 4 Result

### 4.1 IC-ECG findings

**Figure 1:**
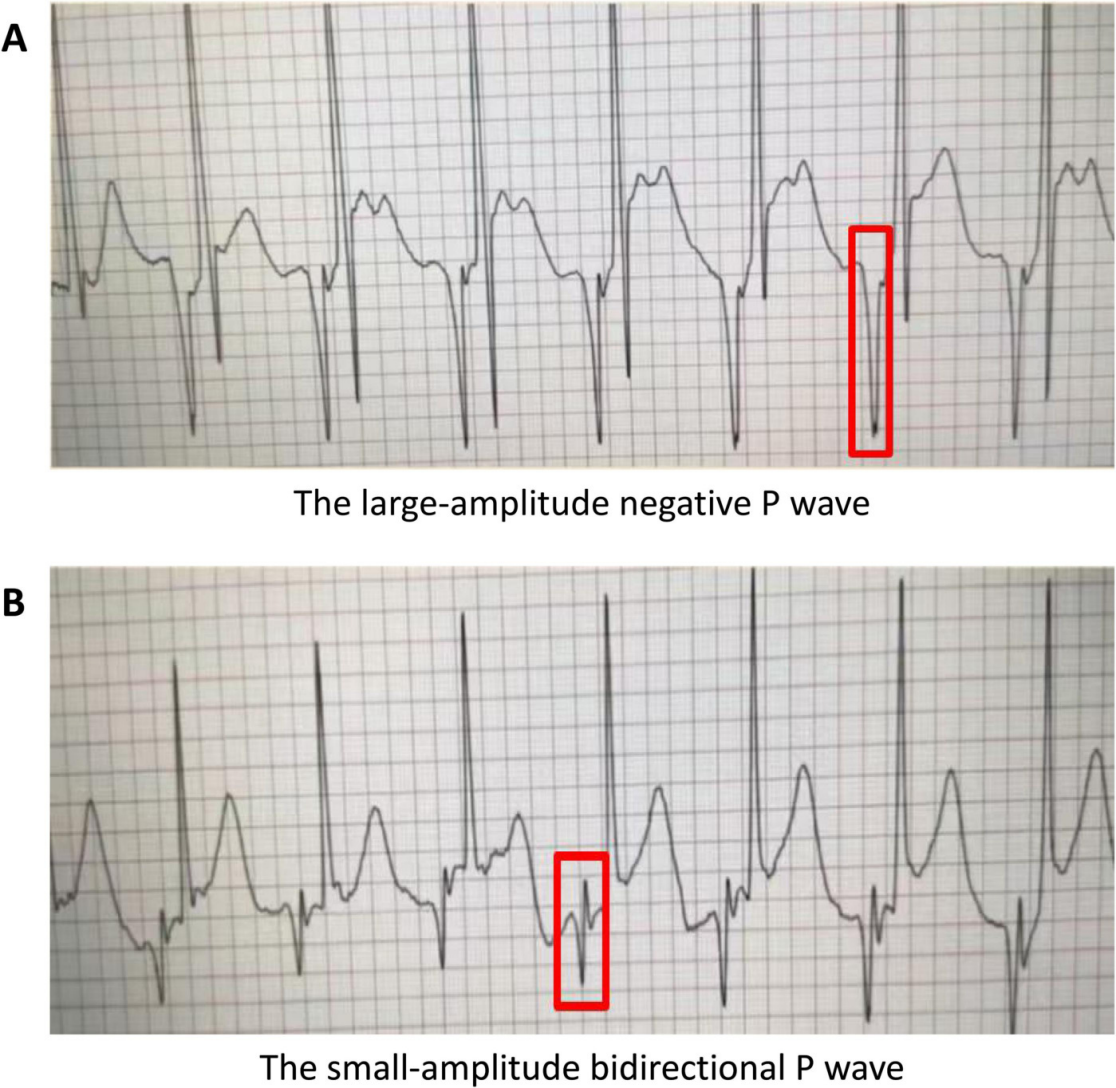
P wave in PLSVC patients during catheterization with IC-ECG.

**Figure 2:**
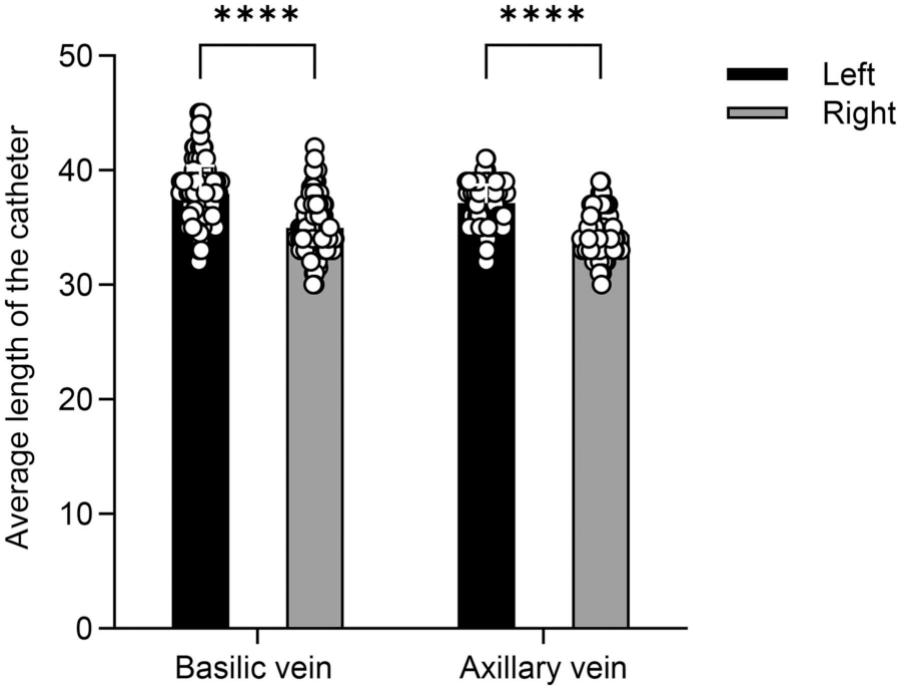
The length of catheterization in patients with RSVC in different venous pathway.

Contrary to the typical positive P wave seen during right superior vena cava (RSVC) catheterization, all three cases exhibited negative P waves (that fell below the isoelectric baseline) once the catheter entered the PLSVC, and its amplitude increasing as the catheter advanced toward the predicted insertion length. With further advancement, the negative P wave gradually decreased in amplitude and eventually transitioned into a low-amplitude biphasic waveform (Figure 1). To verify this characteristic waveform, the catheter tip was advanced beyond the final position only transiently, with the duration of this deeper placement typically lasting less than 2 minutes. Throughout this period, continuous IC-ECG monitoring showed no evidence of arrhythmias (such as premature atrial contractions or atrial fibrillation), and all patients remained asymptomatic. This pattern was reproducible during repeated catheterizations. To avoid excessive tip depth, the catheter was withdrawn by approximately 3 cm from the predicted length based on our empirical findings regarding the length discrepancy between ipsilateral and contralateral venous access routes (Figure 2).

### 4.2 Postoperative X-ray/CT findings

**Figure 3:**
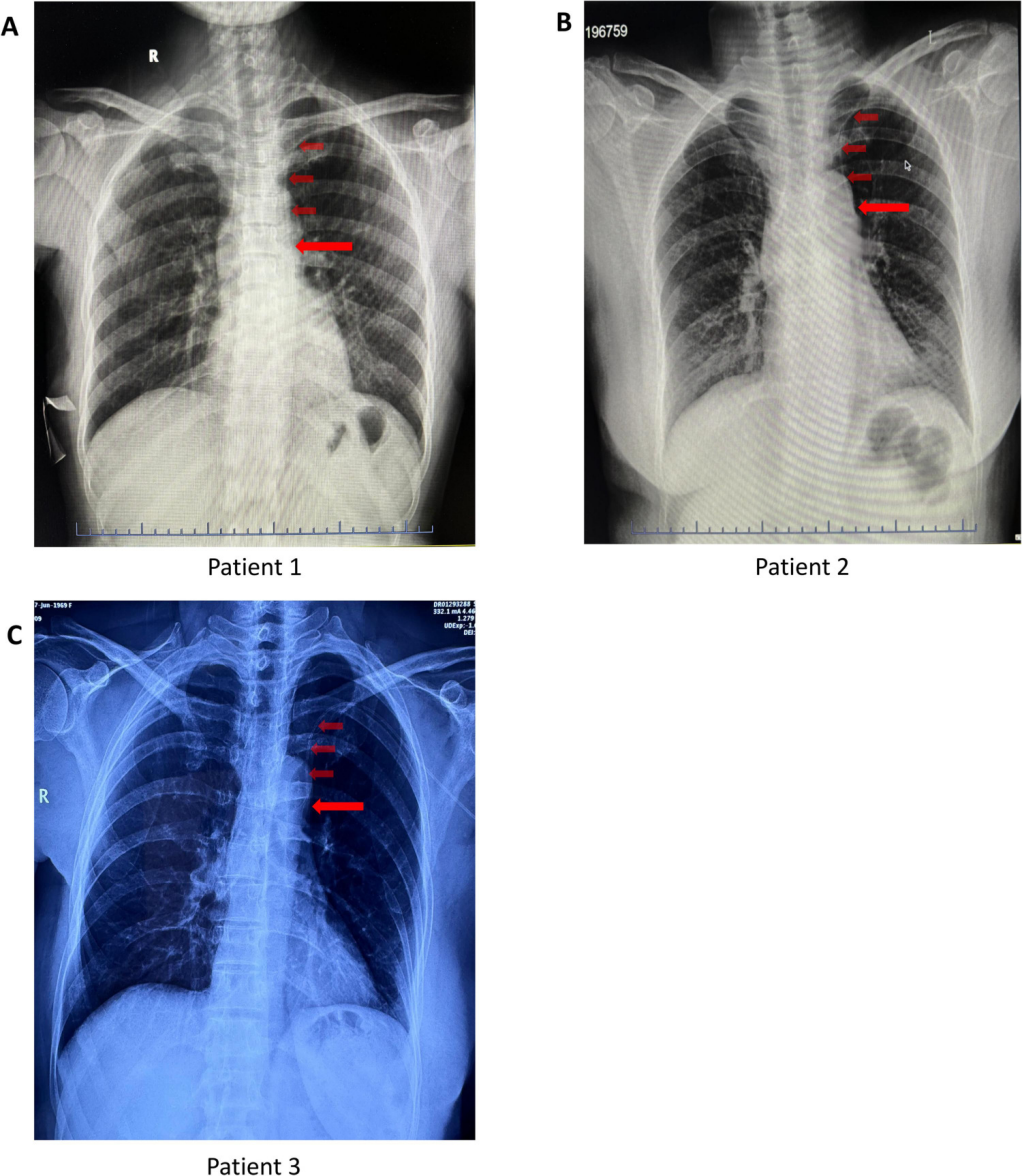
Postoperative chest X-ray of PLSVC patients.

**Figure 4:**
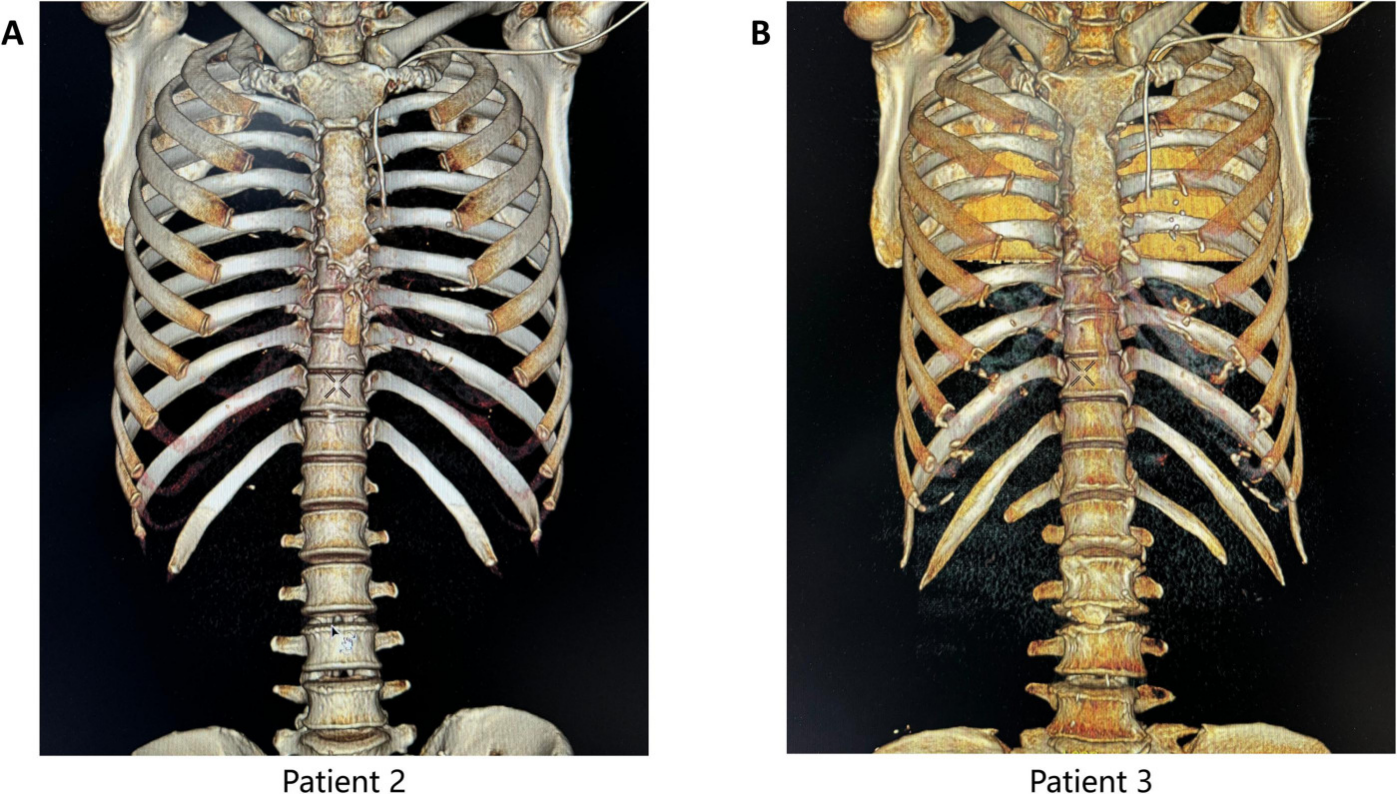
Postoperative 3D CT reconstruction of PLSVC patients.

Chest X-ray examination showed the catheter descending along the left mediastinum instead of the right, with the tip positioned near the T6 level after retraction (Figure 3). Three-dimensional (3D) CT reconstruction in two patients confirmed catheter placement consistently along the left side of the sternum, with the tip at the T6 level —corresponding to the middle to lower third of the PLSVC (Figure 4).

## 5 Discussion

Most patients with PLSVC (up to 90%) have bilateral superior vena cava, among whom 65% lack a bridging innominate vein and the RSVC is usually smaller (9). Based on hemodynamic characteristics, PLSVC can be classified into four types (Table 2). Type I, the most common form (∼90%), is characterized by venous drainage from the PLSVC into the right atrium (RA) via the coronary sinus (CS) without physiological abnormalities. In contrast, Types II-IV involve varying degrees of right-to-left shunt, where venous blood enters the left atrium (LA) directly or indirectly (10), resulting in arteriovenous mixing and clinical manifestations such as cyanosis, which renders catheterization unsuitable.

**Table 2:** Characteristics of the four types of PLSVC.

Type	Anatomy			Blood Flow	Characteristics	Shunt Type	Major Risks
	Origin	Course	Drainage				
I	Confluence of LIJV and LSV	LAG	CS	PLSVC → dilated CS → RA → PC → SC	No obstruction, no abnormal shunt	Left-to-right	·Mild compression of left atrium/left ventricle due to CS dilatation ·Occasionally causing mild reduction in left ventricular diastolic function
II	Same as Type I	Same as Type I	RA (with a tiny channel to LA)	PLSVC → abnormal orifice in RA (minute blood shunts into LA via tiny channel) → LV → SC	Orifice adjacent to LA with a minute shunt; main flow drains into RA, with tiny blood leakage into LA	Right-to-left (minute, occult)	·Occult hypoxemia from long-term micro-shunting ·Easy onset of hypoxia after activity
III	Same as Type I	Same as Type I	LA	PLSVC → LA → LV → SC	If RSVC is absent, all systemic venous blood enters LA	Right-to-left (massive, significant, direct)	·Chronic severe hypoxemia ·Progressively worsening pulmonary hypertension ·Thrombosis
IV	Same as Type I	Mostly runs anterior to LPA	PV/PA	PLSVC → PV/PA → abnormal shunt through PC → LA → LV → SC	PC as intermediate shunt segment, with increased pulmonary perfusion	Right-to-left (indirect, combined with abnormal PC)	·Severe hypoxemia (shunt + abnormal pulmonary perfusion) ·Early-onset & rapidly progressive pulmonary hypertension, leading to right heart failure in short term ·Long-term excessive right heart load causing tricuspid regurgitation and global cardiac enlargement

LIJV, left internal jugular vein; LSV, left subclavian vein; LAG, left atrioventricular groove; CS, coronary sinus; RA, right atrium; PC, pulmonary circulation; SC, systemic circulation; RA, right atrium; LA, left atrium; LV, left ventricle; LPA, left pulmonary artery; PV, Pulmonary vein.

All three cases in our study were ultimately identified as Type I PLSVC. Although this classification was confirmed retrospectively via postoperative CT, our initial procedural strategy was guided by the absence of clinical contraindications like hypoxia. Given that approximately 90% of PLSVC cases are Type I, central venous catheterization is generally feasible and safe for the vast majority of these patients under real-time IC-ECG guidance, provided there is no significant hemodynamic compromise (11, 12). Consequently, IC-ECG can serve as an effective intraoperative screening tool. When a negative P-wave suggests PLSVC, the absence of hypoxia or cyanosis reinforces a high probability of the Type I variant, justifying the continuation of the procedure. However, if such symptoms are present, a non-Type I variant is highly likely, and the procedure should be deferred in favor of preoperative confirmatory imaging to prevent exacerbating physiological abnormalities caused by catheter stimulation. This integrated approach allows for a safer, more nuanced procedural strategy that aligns anatomical probability with real-time electrophysiological monitoring.

PLSVC can be diagnosed using various imaging modalities, each with advantages and limitations. Echocardiography is the most common tool, with PLSVC often suspected when a dilated CS is observed due to increased venous return via the CS (13). This finding is particularly supportive in isolated PLSVC or when blood flow through the vessel is substantial (14). Four-dimensional echocardiography has been reported to provide cost-effective diagnostic utility (15). However, CS dilation is not specific and may occur in other conditions, such as anomalous pulmonary venous connection, pulmonary hypertension, or right heart failure. Therefore, differential diagnosis is essential. Postoperative chest radiography is commonly used to assess the position of central venous catheters (CVCs) or peripherally inserted central catheters (PICCs). A catheter projecting along the left mediastinum on imaging may indicate PLSVC (16). Although this approach enables timely detection of malposition, it often does not permit intraoperative correction without additional intervention. Intraoperative fluoroscopy, by contrast, provides real-time guidance but carries the drawbacks of radiation exposure and increased equipment requirements. Multislice CT and MRI can directly delineate the course and drainage of PLSVC (17). However, because most patients are asymptomatic, these advanced imaging modalities are generally not performed preoperatively in the absence of clinical indications. Surface ECG may also suggest PLSVC, with a positive/negative or entirely negative P wave in lead III predictive of its presence (18), a finding supported by Wang et al. (10). While sensitivity can reach 100%, specificity is lower (≈83%) because other cardiac conditions, such as ectopic pacemakers, may produce similar patterns. Moreover, such subtle changes can be overlooked by non-specialists.

IC-ECG is a simple, safe and recommended method for catheter tip positioning (19). Normally, entry of the catheter into the RSVC produces a progressively taller positive P wave; when the tip passes the sinoatrial node and reaches the mid-RA, a biphasic P wave appears (20). In contrast, entry into PLSVC results in a negative P wave, which increases in amplitude as the catheter advances and may eventually become biphasic (negative-positive) (6, 10, 21), the phenomenon also observed in our cases. Notably, in the study by Wu et al. (20), the biphasic waveform also emerged during catheter withdrawal. Additionally, negative P waves in PLSVC are thought to reflect an opposite electrical vector due to altered conduction pathways (18).

PLSVC is more frequently encountered during left-sided catheterization, but in isolated PLSVC, right-sided access may also enter the anomalous vessel due to RSVC absence. In such cases, predicted insertion length from body surface measurements may overestimate the actual requirement. It is crucial to note that while a negative P-wave is a hallmark finding in our cases, it should be interpreted with caution and is not pathognomonic for PLSVC. Clinicians must consider a broad differential diagnosis, as similar electrophysiological patterns can arise in other contexts. For instance, negative P-waves can occur during standard RSVC catheterization if the tip is advanced too deeply into the RA; notably, these typically resolve upon retraction, a feature that can aid in differentiating them from PLSVC. Other potential causes include the presence of ectopic pacemakers or post-ablation conduction changes (22, 23). Therefore, a negative P-wave should not be viewed in isolation but must be promptly evaluated in conjunction with the patient's medical history and clinical manifestations to ensure an accurate anatomical and functional interpretation.

Safe long-term catheterization in PLSVC requires a strategic approach to access selection. Priority must be given to minimizing the risks of infection, thrombosis, and catheter dislodgment. In this regard, implantable venous ports have been demonstrated to be superior to PICCs for long-term use (24, 25). Furthermore, considering the necessity for less frequent maintenance and the preservation of patient quality of life, thoracic implantable ports remain the preferred choice over both PICCs and femoral venous access. However, anatomical constraints must guide the final decision. The catheter-to-vessel diameter ratio should be <45% to mitigate the risk of venous thromboembolism (VTE) risk (26, 27). A narrow PLSVC diameter may predispose the patient to residual catheter retention or occlusion (28), so alternative sites should be considered in such cases. Furthermore, catheterization in PLSVC with LA drainage is generally contraindicated due to the potential for exacerbating cyanosis and other systemic adverse effects (29). Optimal tip positioning should avoid the CS, as malposition may cause early postoperative facial edema and thrombosis (30), exacerbate CS dilation, and trigger arrhythmias by compressing conduction tissue (31, 32). Positioning in the mid- to lower third of the PLSVC, which typically corresponds to the T6 vertebral level, is recommended (19, 26).

The selection of the T6 level as the target for the catheter tip is supported by critical clinical and physiological considerations. Anatomically, this level represents a hemodynamic spot characterized by stable and adequate blood flow velocity. This facilitates the rapid dilution of chemotherapy drugs, thereby minimizing the risk of intimal irritation and venous thrombosis. From a safety perspective, maintaining the tip at T6 ensures it remains superior to the CS ostium. This is essential because mechanical contact or malposition within the CS can lead to CS enlargement, localized flow obstruction (potentially causing postoperative facial edema), and, most significantly, the compression of adjacent cardiac conduction tissues, which may trigger arrhythmias such as atrial fibrillation. Thus, the T6 level provides an optimal balance between ensuring efficient infusion and maximizing procedural safety.

Recent literature has described varied approaches to achieving accurate tip positioning in PLSVC (Table 1). Chest radiograph or fluoroscopy remains standard and intuitive method, but it not only carries risks of radiation exposure but also requires a higher level of operating room equipment and configuration. IC-ECG is increasingly adopted as an alternative, although a unified standard has not yet been established. Different strategies have been reported: tip placement at the maximum positive P wave in lead II (21) and fixation at a P-to-R ratio of approximately 50% of the maximum negative P wave (10). In neonatal PLSVC catheterization, a more meticulous stepwise approach has been described (20). Upon detection of inverted P waves, the catheter was retracted in 0.5-cm increments, with this standardized retraction protocol maintained until the electrophysiological pattern transitioned to biphasic P waves. After complete resolution of the aberrant P-wave morphology, an additional 0.5 cm retraction was performed to confirm proper intravascular positioning within the vena cava before final catheter securement. In this method, the final position of the catheter tip is independent of the absolute amplitude of the P wave.

Our technique integrates P-wave morphology with predicted catheter length from surface measurements. When a negative P wave was observed at the predetermined insertion length, the catheter was withdrawn by about 3 cm, and this adjusted position was confirmed as the final tip location. This approach was informed by our prior findings (data collected from January 2022 to April 2025) that ipsilateral punctures (e.g., left-sided punctures into PLSVC or right-sided punctures into RSVC) consistently require shorter catheter lengths than contralateral punctures. The mean difference was approximately 3 cm, with measured average reductions of 2.980 cm in the axillary vein and 2.762 cm in the basilic vein (Figure 2). This discrepancy arises because the measured value often corresponds to the expected distance for a left-sided puncture into the (a contralateral route), whereas the actual pathway in PLSVC is ipsilateral and thus shorter.

Accordingly, once PLSVC is identified, the 3 cm withdrawal serves as a practical intraoperative reference based on these empirical observations. However, while this fixed adjustment proved effective in our cases, it should be noted that other strategies—such as percentage-based corrections or anatomy-adjusted formulas—remain equally plausible and may offer advantages in patients with extreme body habitus. Individual anatomical variations may alter the actual discrepancy between these routes; therefore, while the 3 cm retraction provides an efficient intraoperative guide, the final confirmation of catheter tip position must still rely on imaging evaluation. Currently, due to the limited sample size of PLSVC cases, we propose this 3 cm withdrawal as a hypothesis-generating guide rather than a definitive protocol. The optimal adjustment method remains a subject for further investigation, and our approach requires validation through larger, multi-center cohorts.

While IC-ECG is not intended to replace cross-sectional imaging (such as CT) for the definitive anatomical confirmation of PLSVC, it offers significant incremental value by providing real-time intraoperative guidance. In this study, the observation of a characteristic negative P-wave via IC-ECG served as an early warning sign, triggering immediate suspicion of PLSVC. This allowed for an instantaneous pullback strategy to prevent deep catheter malposition during the primary procedure. Consequently, although definitive diagnosis was confirmed by postoperative CT, the preemptive adjustment guided by IC-ECG effectively obviated the need for secondary repositioning surgeries, demonstrating its critical utility in enhancing procedural safety and efficiency.

Potential complications, which are particularly pertinent in the context of PLSVC, include arrhythmias (notably atrial fibrillation) due to mechanical stimulation of the conduction system near the CS, vascular injury during advancement through anomalous pathways, early thrombus formation (33), and long-term catheter fixation with the risk of breakage upon removal (34); notably, none were encountered in our cases. However, while catheterization is often successful in asymptomatic Type I cases, it should not be portrayed as a straightforward procedure. Catheter placement in PLSVC, particularly when draining into the CS, carries specific clinical risks, including CS enlargement, potential venous thrombosis, and early postoperative facial edema due to local venous obstruction.

To optimize procedural safety and mitigate these risks, several precautions are essential. First, the catheter-to-vessel diameter ratio should be maintained at less than 45% to minimize the risk of venous thromboembolism. Second, IC-ECG should be utilized to ensure the catheter tip is positioned in the mid-to-lower third of the PLSVC, thereby avoiding direct entry into the CS. Should non-Type I anatomy be suspected, or if the PLSVC diameter is deemed insufficient for safe catheter accommodation, the procedure must be deferred. In such scenarios, clinicians should systematically consider alternative access strategies, such as right-sided venous access (if the RSVC is present), femoral venous access, or selecting a different puncture site to avoid complications associated with anomalous left-sided drainage.

## 6 Conclusion

PLSVC is a rare vascular anomaly that can pose significant challenges for catheter placement, particularly in patients with right breast cancer requiring left-sided vessel access. IC-ECG guidance is useful in this setting, as the appearance of a negative P wave reliably indicates entry into the PLSVC. Upon identification of PLSVC, withdrawing the catheter by approximately 3 cm from the predicted insertion length immediately can serve as a hypothesis-generating reference to prevent excessive catheter tip dept. Although this approach provides a practical starting point for adjustment, it remains based on limited empirical experience, and further large-scale studies are warranted to validate the optimal management of catheter placement in this patient population. Transient deep catheter positioning, even briefly, may carry risks, and the morphology of the P wave at different positions warrants ongoing observation. Therefore, further investigation into the safety of the procedure is warranted, including potential complications, recommended preventive measures, and alternative access strategies when such anatomical variants are suspected or confirmed. Careful monitoring and individualized assessment remain essential to ensure the safety of catheterization in patients with PLSVC.

## Data Availability

The original contributions presented in the study are included in the article/supplementary material, further inquiries can be directed to the corresponding author.
